# *Magnolia kobus* Extract Suppresses *Porphyromonas gingivalis* LPS-Induced Proinflammatory Cytokine and MMP Expression in HGF-1 Cells and Regulates Osteoclastogenesis in RANKL-Stimulated RAW264.7 Cells

**DOI:** 10.3390/cimb45060310

**Published:** 2023-06-03

**Authors:** Hae Jin Lee, So Jung Lee, Sung Kwon Lee, Bong Keun Choi, Dong Ryung Lee, Ju-Hyoung Park, Joa Sub Oh

**Affiliations:** 1 NUON Co., Ltd., Jungwon-gu, Seongnam-si 13201, Republic of Korea; hjlee@nuon.kr (H.J.L.); sjl4859@nuon.kr (S.J.L.); sklee@nuon.kr (S.K.L.); cbcbcbk@nuon.kr (B.K.C.); drlee@nuon.kr (D.R.L.); 2 College of Pharmacy, Dankook University, Cheonan 31116, Republic of Korea; jhp0607@dankook.ac.kr

**Keywords:** *Magnolia kobus*, periodontitis, gingival tissue destruction, inflammation, ECM degradation, bone resorption, human gingival fibroblast, osteoclast

## Abstract

Clinical prevention is of utmost importance for the management of periodontal diseases. Periodontal disease starts with an inflammatory response in the gingival tissue, and results in alveolar bone destruction and subsequent tooth loss. This study aimed to confirm the anti-periodontitis effects of MKE. To confirm this, we studied its mechanism of action using qPCR and WB in LPS-treated HGF-1 cells and RANKL-induced osteoclasts. We found that MKE suppressed proinflammatory cytokine protein expression by inhibiting the TLR4/NF-κB pathway in LPS-PG-induced HGF-1 cells and blocking ECM degradation by regulating the expression of TIMPs and MMPs. We also confirmed that TRAP activity and multinucleated cell formation were reduced in RANKL-stimulated osteoclasts after exposure to MKE. These results were confirmed by inhibiting TRAF6/MAPK expression, which led to the suppression of *NFATc1*, *CTSK*, *TRAP*, and MMP expression at the gene and protein levels. Our results confirmed that MKE is a promising candidate for the management of periodontal disease based on its anti-inflammatory effects and inhibition of ECM degradation and osteoclastogenesis.

## 1. Introduction

Periodontal disease is an inflammatory condition caused by Gram-negative bacteria and characterized by soft tissue destruction in the gingiva and alveolar bone resorption [1]. Anaerobic Gram-negative bacterium *Porphyromonas gingivalis* (Pg) is the most significant periodontal pathogen involved in the progression of periodontal diseases. Lipopolysaccharide (LPS), a bacterial endotoxin secreted by Pg, activates Toll-like receptor (TLR) 4 and accelerates the development of periodontitis by increasing the inflammatory response and bone resorption [2,3,4,5,6]. Osteoclasts degrade the bone tissue through a process known as bone resorption. In periodontitis, osteoclasts degrade the bone supporting the teeth, leading to tooth loss due to the lack of alveolar bone support [7,8].

The most abundant human gingival fibroblasts in gingival connective tissue play a critical role in regulating homeostasis and the remodeling of periodontal tissue [9,10]. HGF-1 cells react with periodontal pathogenic bacteria and lipopolysaccharide (LPS), initiating an inflammatory response. LPS binds to TLR4 and causes downstream IκBα degradation, where free NF-κB is translocated into the nucleus to encode inflammatory mediators or proinflammatory cytokines such as IL-6, IL-1, and TNF-α [2,3,6,11]. High-mobility group box 1 (HMGB1) is a non-histone chromosomal protein that extensively binds to DNA and regulates transcription. HMGB1 is secreted in response to LPS or oxidative stress stimulation and binds to TLR4, increasing the amount of proinflammatory mediators released [12,13].

Cellular adhesion molecules (CAM) are proteins that can adhere to the gums, junctional epithelium, and gingival sulcus surfaces. They are important components in the connection between cells and the extracellular matrix (ECM). Vascular cell adhesion molecule 1 (VCAM-1) and intercellular adhesion molecule 1 (ICAM-1) belong to the Ig superfamily and are known to be expressed in leucocytes, monocytes, fibroblast, and synovial cells. Proinflammatory cytokines promote monocyte adhesion and leukocyte recruitment by increasing the expression of VCAM-1 and ICAM-1, which are involved in controlling the development and progression of periodontal diseases [14,15,16,17]. HGF-1 can also produce ECM components, such as collagen and elastin, and may be involved in maintaining or destroying periodontal tissue by producing and secreting matrix metalloproteinases (MMPs) in response to LPS [4]. MMPs are proteases that degrade the ECM and have a catalytic domain containing a zinc atom in its active site [18]. Tissue inhibitors of metalloproteinase (TIMPs) are proteins composed of 184–194 amino acids known to inhibit MMP activity. Periodontal disease occurs when the balance between MMPs and TIMP is disrupted. Excessive MMP expression stimulates ECM decomposition, leading to the loss of connective tissue adhesion, destruction of the supporting bone, further tooth loss, and various inflammatory diseases [4,19,20].

Osteoclasts derived from hematopoietic precursor cells are large multinucleated cells in the bone marrow. Their survival, differentiation, and proliferation are regulated by receptor activator of nuclear factor-κB ligand (RANKL) and macrophage colony-stimulating factor (M-CSF) [7,21]. The binding of RANK to RANKL is mediated by the recruitment of TRAF adapter proteins [22]. RANKL signaling through TRAF6 activates several downstream signaling pathways, including MAPKs [23,24], which ultimately induces the expression of nuclear factor of activated T cells c1 (NFATc1). NFAT family member NFATc1 is an important regulator of osteoclastogenesis that upregulates cathepsin K (CTSK), tartrate-resistant acid phosphatase (TRAP), and MMPs, which are osteoclast-specific genes involved in osteoclast formation and function [25,26,27].

*Magnolia flos* has a long history as a Chinese medicinal herb for treating various clinical symptoms. *Magnolia kobus* is a member of *Magnolia* spp., and its dried flower buds have been used as medicine for their anti-inflammatory and anti-allergic properties and for headache, toothache, and nasal congestion [28,29,30]. In previous studies, the inhibitory effect of *Magnolia flos* on histamine release in rat peritoneal mast cells has been confirmed. Additionally, a study has reported the anti-inflammatory activity of the MeOH extract derived from *Magnolia kobus* stem bark. However, studies investigating the applicability of MKE to gingivitis/periodontitis have not yet been conducted [31,32]. In our previous study, we examined the changes in the inflammatory response to *Magnolia kobus* and its active compound, magnolin, in LPS-induced RAW264.7 cells [33]. Nevertheless, the precise mechanisms underlying the effect of *Magnolia kobus* extract (MKE) on gingival destruction and bone resorption have not yet been studied. Therefore, in the present study, we investigated the factors related to inflammation and ECM degradation in gingival fibroblasts induced by LPS-PG, a causative agent of periodontitis and factors related to RANKL-induced osteoclastogenesis in osteoclasts.

## 2. Materials and Methods

### 2.1. Preparation of Magnolia kobus Extract (MKE)

The aqueous *Magnolia kobus* (MK) extract (MKE) was obtained from NUON Co., Ltd. (Seongnam, Republic of Korea). As described in a previous study [33], cleanly dried MK was extracted with water. The prepared extract was produced by filtering, concentrating, and drying, and only MKE standardized to approximately 1.5% magnolin content was used in the experiments.

### 2.2. Cell Culture

The human gingival fibroblast (HGF-1) and murine macrophage RAW264.7 cell lines (American Type Culture Collection, Rockville, MD, USA) were maintained in Dulbecco’s modified Eagle’s medium (DMEM; Gibco, Grand Island, NY, USA) supplemented with 10% fetal bovine serum (FBS; Gibco) and 1% penicillin-streptomycin (Gibco) in a humidified incubator with 5% CO_2_ at 37 °C. HGF-1 cells were subcultured twice or thrice a week and used only up to passage 10 for the experiments. RAW 264.7 cells were seeded in 6- well plate at a density of 10^5^ cells/well and incubated with α-minimal essential medium (α-MEM) supplemented with 100 ng/mL RANKL (Peprotech Inc., Rocky Hill, NJ, USA) for differentiation into osteoclasts.

### 2.3. Cell Viability Measurement

Cell viability was measured using a 3-(4,5-dimethylthiazol-2-yl)-2,5-diphenyl tetrazolium bromide (MTT; Duchefa Biochemistry, Haarlem, The Netherlands) assay to confirm the toxicity of MKE in HGF-1 cells and RANKL-induced osteoclasts. Briefly, cells were seeded and maintained for 24 h. HGF-1 cells were treated with MKE (0–10 μg/mL) with or without LPS-PG (1 μg/mL) for 24 h. RAW264.7 cells were incubated with MKE (0–100 μg/mL) and 100 ng/mL RANKL for 5 days before adding an MTT solution (0.5 mg/mL) followed by further incubation for 4 h. Afterward, dimethyl sulfoxide (DMSO; Wako, Osaka, Japan) was added and measured at 570 nm absorbance using a microplate reader (Tecan, Mannedort, Switzerland).

### 2.4. TRAP Staining and Activity Analysis

RAW264.7 cells were seeded in 96-well plates containing α-MEM supplemented with 100 ng/mL RANKL at a density of 2 × 10^3^ cells/well. The cells were treated with MKE for five days. TRAP staining was performed using a TRAP staining reagent containing 5 mg naphthol AS-MX phosphate (Sigma-Aldrich, St. Louis, MO, USA), 500 μL of N,N-dimethylformamide (Wako), and 30 mg Fast red violet LB salt (Sigma-Aldrich) in 50 mL TRAP staining buffer. The TRAP staining buffer was prepared using 0.11% citric acid (Sigma-Aldrich), 14 mmol of sodium acetate trihydrate (Wako), and 10 mmol of sodium (+) tartrate dihydrate (Wako) in distilled water. Cells were fixed with 4% paraformaldehyde, washed with PBS, and 50 μL/well of TRAP staining reagent was added to stain the cells. TRAP-positive cells were dark red and cells containing three or more nuclei were classified as osteoclasts. TRAP activity was measured spectrophotometrically in 50 mM citrate buffer containing 10 mM sodium tartrate and 6 mM p-nitrophenyl phosphate (Sigma-Aldrich). Briefly, 100 μL of buffer was added to each well and reacted with 85 μL of 0.1 N NaOH after 20 min. The absorbance of the supernatant was measured at 405 nm using a microplate reader (Tecan).

### 2.5. RNA Extraction and qPCR

Total RNA from MKE-treated HGF-1 cells with or without LPS-PG and RAW264.7 cells treated with or without RANKL were isolated using an RNeasy Mini kit (Qiagen GmbH, Hilden, Germany). Complementary DNA (cDNA) was synthesized using an All-in-one 5x cDNA Master mix (CellSafe, Yongin, Republic of Korea) according to the manufacturer’s instructions and reacted at 25 °C for 5 min, 42 °C for 15 min, and 85 °C for 5 s. The synthesized cDNA was then reacted with SYBR Green Master Mix (Elpis Biotech, Daejeon, Republic of Korea) for qPCR, and the primer information is shown in Table 1. The gene expression levels were determined from the 2^−ΔΔCq^ method and automatically calculated using LightCycler 96 Software 1.1 (Roche Diagnostics, Basel, Switzerland). Relative gene expression levels were normalized and quantified using GAPDH.

### 2.6. Western Blot Analysis

HGF-1 cells were incubated in 6-well plates containing different concentrations of MKE, with or without LPS-PG, for 24 h. Similarly, RAW264.7 cells were incubated in 6-well plates containing different concentrations of MKE with RANKL for 5 days. The cells were washed three times with PBS, CelLytic buffer (Sigma-Aldrich) was added, and the cells were scraped. The cell lysate was centrifuged at 13,000 rpm for 15 min at 4 °C and the protein concentration was quantified using a Bradford assay (Bio-Rad Laboratories, Hercules, CA, USA). The proteins (20 μg) from each lysate were separated using 8–12% sodium dodecyl sulfate-polyacrylamide gel electrophoresis (SDS-PAGE) and electrotransferred to an Immobilon-P membrane (Millipore, Bedford, MA, USA). The membranes were blocked with 5% skim milk for 30 min and washed three times with Tris-buffered saline containing 0.5% Tween-20 (TBS-T) for 30 min. The washed membranes were incubated with a 1:500–1000 dilution of specific primary antibody at 4 °C overnight and then incubated with the corresponding horseradish peroxidase-conjugated anti-mouse or anti-rabbit secondary antibodies for 1 h. Signals from the blots were detected using an ECL solution (GenDEPOT, Barker, TX, USA) and measured using a LuminoGraph (Atto, Tokyo, Japan). The visualized images and intensities were quantified using ImageJ software (NIH, Bethesda, MD, USA) and normalized to that of β-actin.

### 2.7. Statistical Analysis

All presented data are expressed as mean ± standard deviation, and statistical analysis was performed using SPSS 12.0.0 (IBM Co., Armonk, NY, USA). Statistical differences between groups were analyzed using Student’s t-test and statistical significance was set at *p* < 0.05.

## 3. Results

### 3.1. Effects of MKE on Cell Viability in HGF-1 Cells and RANKL-Induced Osteoclasts

**Figure 1:**
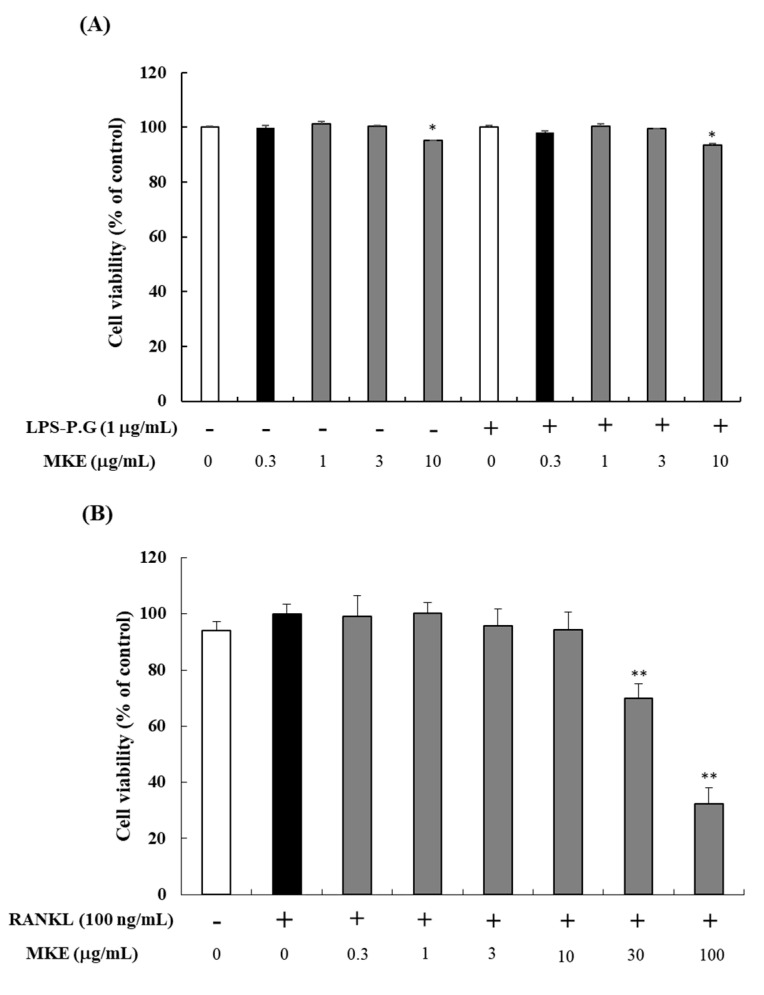
(**A**) Effects of *Magnolia kobus* extract (MKE) treatment (0.3, 1, 3, or 10 μg/mL) on HGF-1 cell viability. (**B**) Effects of MKE treatment (0, 0.3, 1, 3, 10, 30, or 100 μg/mL) on RAW264.7 cell viability. HGF-1 cells were incubated with MKE for 24 h with or without LPS-PG (1 μg/mL), while RAW264.7 cells were maintained with RANKL (100 ng/mL) for 5 days. After incubation, the cell viability was assessed using an MTT assay. The experiments (*n* = 3) were conducted three times independently and the values are expressed as mean ± standard deviation. * *p* < 0.05 and ** *p* < 0.01 vs. the MKE 0 μg/mL group.

The cell viability of MKE on HGF-1 cells was evaluated using the MTT assay (Figure 1A). HGF-1 cells were incubated with MKE (0, 0.3, 1, 3, or 10 μg/mL) for 24 h. Treatment with 10 μg/mL MKE showed significant cell toxicity (* *p* < 0.05), and treatment with 10 μg/mL MKE and LPS-PG (1 μg/mL) also significantly decreased cell viability (* *p* < 0.05). Therefore, MKE concentrations of 0.3–3 μg/mL were used in subsequent experiments. Likewise, RAW264.7 cells were incubated with RANKL and MKE (0, 0.3, 1, 3, 10, 30, or 100 μg/mL) for 5 days (Figure 1B). The viability of RAW264.7 cells significantly decreased at MKE concentrations of 30 and 100 μg/mL (** *p* < 0.01). Therefore, MKE concentrations between 1 and 10 μg/mL were used in subsequent experiments with RAW264.7 cells.

### 3.2. MKE Reduces Gingival Inflammation by Regulating the TLR4/NF-κB Pathway in LPS-PG-Treated HGF-1 Cells

**Figure 2:**
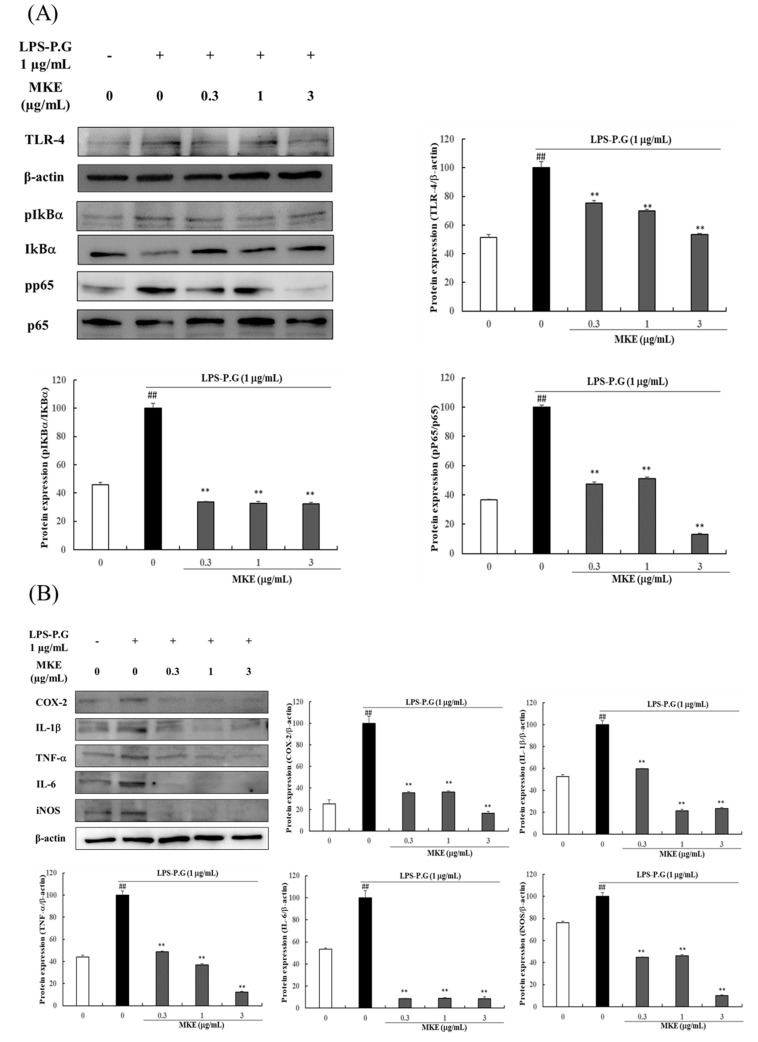

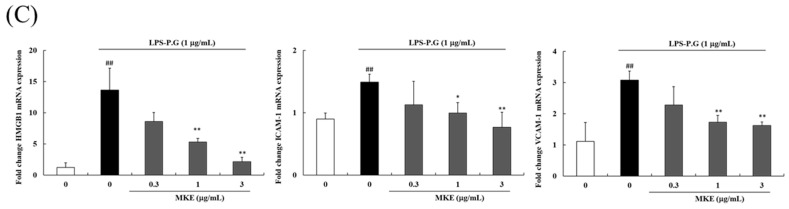
Effects of *Magnolia kobus* extract (MKE) treatment on the expression of the (**A**) TLR4/NF-κB pathways, (**B**) inflammatory cytokines (COX-2, IL-1β, TNF-α, IL-6, and iNOS), and (**C**) the mRNA levels of *HMGB1*, *ICAM-1* and *VCAM-1* in 1 μg/mL LPS-PG-stimulated HGF-1 cells. HGF-1 cells were incubated in the presence of LPS-PG (1 μg/mL) and MKE (0.3, 1, or 3 μg/mL) for 15 min or 24 h. The mRNA levels were assessed via RT-PCR and protein levels were detected using a Western blot assay. The protein band density was determined using ImageJ software. Each experiment was repeated three times (*n* = 3) and each value was normalized to that of β-actin and GAPDH. Data are represented as mean ± standard deviation. ^##^ *p* < 0.01 vs. the vehicle control group; * *p* < 0.05 and ** *p* < 0.01 vs. the LPS-PG control group.

TLR-4 is known to activate the NF-κB pathway by initially recognizing LPS. LPS treatment significantly increased the expression level of TLR4 (^##^ *p* < 0.01) and markedly enhanced the phosphorylation of IκBα and p65 protein (^##^ *p* < 0.01) compared to the vehicle control group. However, the groups treated with MKE (0.3–3 μg/mL) dose-dependently suppressed TLR4 expression levels by 24.70 ± 1.68%, 30.14 ± 0.96%, and 46.55 ± 0.71%, respectively. Similarly, the phosphorylation of IκBα protein was diminished in a dose-dependent manner (66–67%) in the MKE (0.3–3 μg/mL) treatment groups compared to the LPS-only treated group. MKE also significantly reduced p65 phosphorylation by 63.36–83.61% compared to the LPS-only treated group (Figure 2A).

### 3.3. MKE Inhibits LPS-PG-Stimulated Gingival Inflammation and Decreases HMGB1, ICAM-1, and VCAM-1 Expression in HGF-1 Cells

After 24 h of LPS-PG treatment, COX-2, IL-1β, TNF-α, IL-6, and iNOS protein expression levels were significantly increased (^##^ *p* < 0.01) compared to those in the LPS-PG-untreated control cells. However, MKE treatment (0.3–3 μg/mL) showed a significant decrease in COX-2 expression levels by 40.27 ± 0.70%, 78.55 ± 0.76%, and 76.45 ± 1.68%, respectively. MKE treatment also reduced IL-1β levels by 51.12 ± 1.50%, 63.21 ± 1.68%, and 87.8 ± 0.41%, respectively, compared to the LPS-PG-treated control. TNF-α protein expression showed a similar reduction effect, up to 91%, after treatment with 0.3–3 μg/mL MKE. Furthermore, MKE treatment (0.3–3 μg/mL) significantly decreased IL-6 expression levels by 55.34 ± 4.41%, 53.86 ± 4.21%, and 90.01 ± 1.38%, respectively, while iNOS protein expression resulted in a significant decrease of 72.90 ± 0.38%, 61.66 ± 0.89%, and 67.54 ± 1.58%, respectively (Figure 2B). In addition, cytokine levels were induced by LPS-PG treatment. We observed significant increases in HMGB1, ICAM-1, and VCAM-1 mRNA expression levels, by 13.64-fold, 1.49-fold, and 3.07-fold, respectively, compared to the vehicle-treated control (all ^##^ *p* < 0.01). However, MKE treatment (1 and 3 μg/mL) decreased the mRNA expression levels in the range of 61.29–84.39% for *HMGB1*, 33.42–48.59% for *ICAM-1*, and 43.79–47.42% for *VCAM-1* in a concentration-dependent manner (Figure 2C).

### 3.4. MKE Downregulates MMP Expression and Upregulates TIMP Expression in LPS-PG-Stimulated HGF-1 Cells

**Figure 3:**
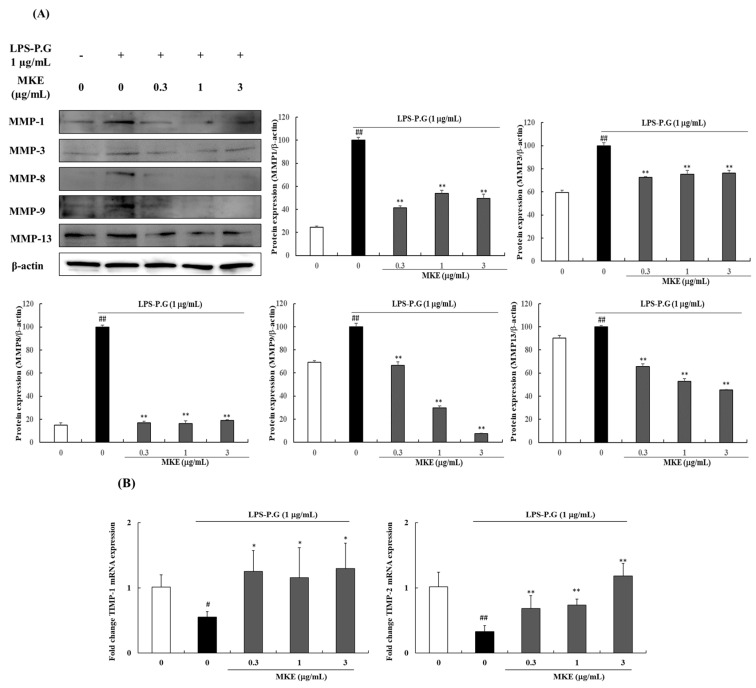
Effects of *Magnolia kobus* extract (MKE) treatment (0.3, 1, or 3 μg/mL) on (**A**) the protein expression of matrix metalloproteinase (MMP)-1, 3, 8, 9, and 13 and (**B**) mRNA expression levels of tissue inhibitors of metalloproteinase (TIMP)-1 and 2 in LPS-PG-stimulated HGF-1 cells. HGF-1 cells were incubated in the presence of LPS-PG (1 μg/mL) and MKE (0.3, 1, or 3 μg/mL) for 24 h. The mRNA levels were assessed via RT-PCR and protein levels were detected using a Western blot assay. The protein band density was determined using ImageJ software. Each experiment was repeated three times (*n* = 3) and each value was normalized to that of β-actin and GAPDH expression. Data are represented as mean ± standard deviation. ^#^ *p* < 0.05 and ^##^ *p* < 0.01 vs. the vehicle control group; * *p* < 0.05 and ** *p* < 0.01 vs. the LPS-PG control group.

MMPs which are known to play an important role in ECM degradation, were significantly increased (^##^ *p* < 0.01) in the LPS-PG-treated group compared with the vehicle-treated control group (LPS-PG-untreated). The increased MMP-1 protein expression level was reduced by 58.67 ± 1.72%, 46.21 ± 2.71%, and 50.62 ± 3.85% compared with the LPS control group after treatment with MKE (0.3, 1, and 3 μg/mL, respectively). Similarly, MMP-3 and MMP-8 protein expression showed a 23.70–27.48% and 80.92–83.76% reduction, respectively, compared to the LPS control group after treatment with 0.3–3 μg/mL MKE. Similarly, MMP-9 protein levels decreased by 33.49 ± 2.93%, 70.12 ± 1.60%, and 92.35 ± 0.22%, while MMP-13 protein expression decreased by 34.37 ± 2.32%, 47.04 ± 2.24%, and 54.71 ± 0.57%, respectively, compared to the LPS-only treated group in a concentration-dependent manner (Figure 3A). The expression of TIMPs, which inhibit the activity of MMPs, showed a significant decrease compared to the vehicle control (LPS-PG-untreated) following LPS-PG treatment. The decreased TIMP-1 gene expression increased (* *p* < 0.05) by 1.15–1.29-fold following MKE treatment, while TIMP-2 expression also recovered (** *p* < 0.01), similar to the control group after treatment with 1 μg/mL MKE (Figure 3B). These results show that MKE treatment regulates the expression of factors related to inflammatory responses and ECM degradation, as well as the TLR4/NF-κB pathway, in HGF-1 cells.

### 3.5. MKE Inhibits Osteoclastogenesis in RAW264.7 Cells Induced by RANKL

**Figure 4:**
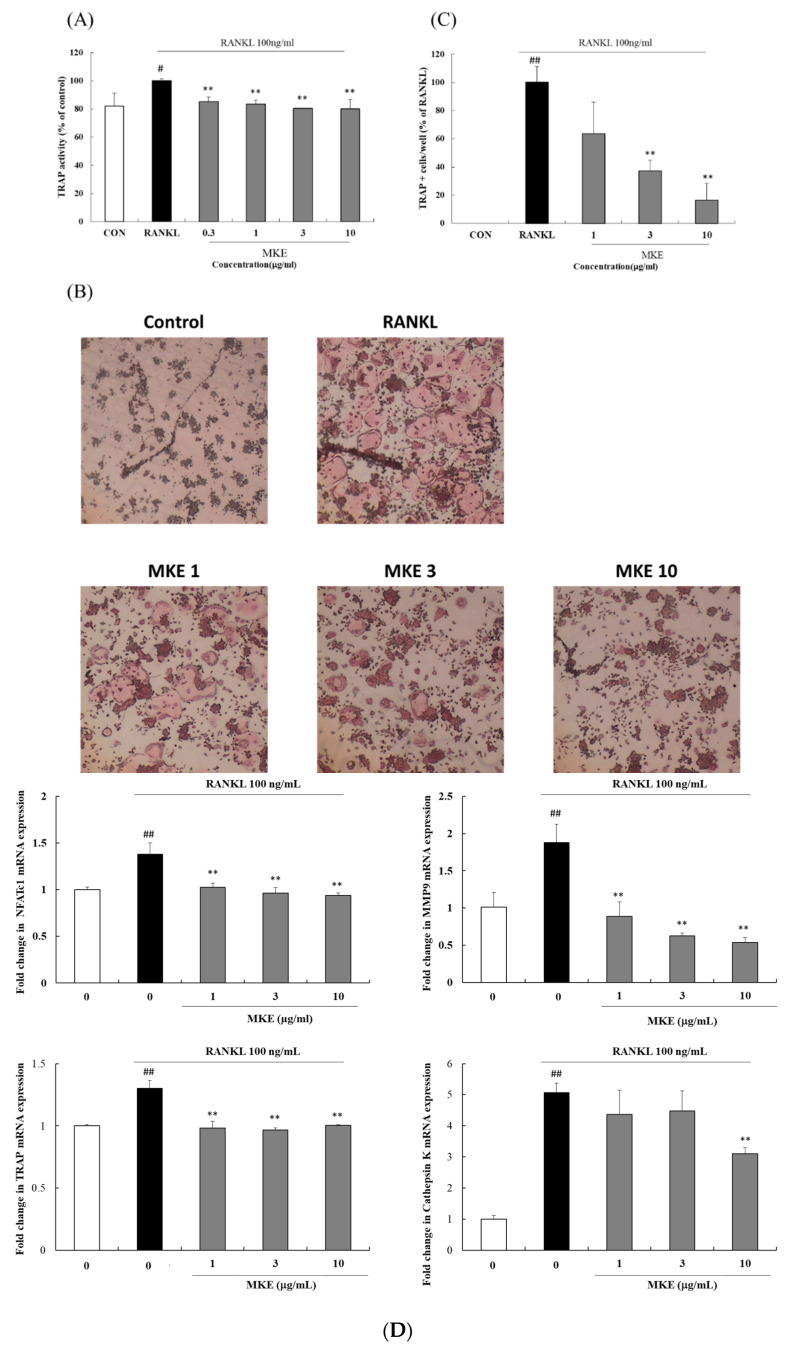

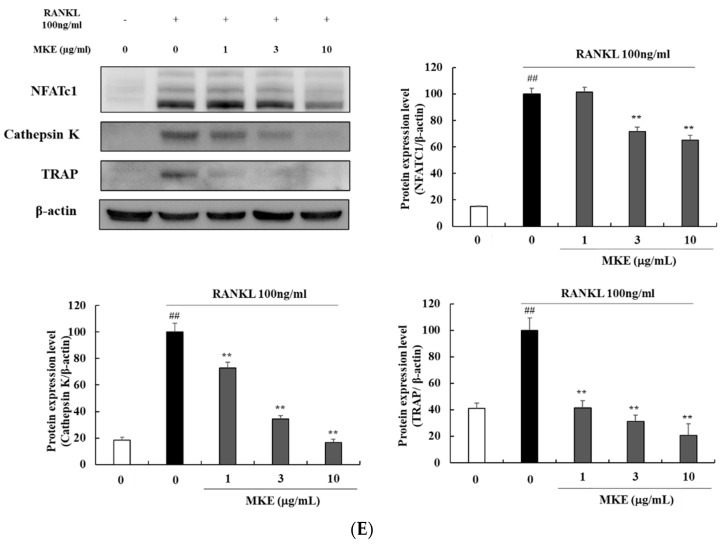
Effect of *Magnolia kobus* extract (MKE) (0.3, 1, 3, or 10 μg/mL) treatment on osteoclast differentiation in RAW264.7 cells. MKE was co-treated with RANKL for 5 days and (**A**) TRAP activity and (**B**) TRAP staining were performed. (**C**) TRAP-positive cells in each well were observed under a microscope and counted. TRAP-positive cells were stained red and contained at least three nuclei. (**D**) The gene expression levels of NFATc1, MMP9, TRAP, and Cathepsin K were detected via RT-PCR. (**E**) The protein expression levels of NFATc1, Cathepsin K, and TRAP were detected via Western blot. The protein band density was determined using ImageJ software. Each experiment was repeated three times (*n* = 3) and each value was normalized to that of β-actin and GAPDH. Data are represented as mean ± standard deviation. ^#^ *p* < 0.05 and ^##^ *p* < 0.01 vs. the vehicle control group; ** *p* < 0.01 vs. the RANKL control group.

RAW264.7 cells were exposed to RANKL to induce their differentiation into TRAP-positive cells. Next, RAW264.7 cells were stained using a TRAP staining reagent to test the effect of MKE on osteoclastogenesis. The TRAP-positive cell numbers were significantly increased with RANKL treatment but decreased by 62.81 ± 7.44% and 83.47 ± 11.72% after treatment with 3 and 10 μg/mL of MKE, respectively (Figure 4C). In addition, TRAP activity (Figure 4A) was significantly increased by RANKL treatment and decreased by 14.91 ± 3.33%, 16.57 ± 2.63%, 19.53 ± 0.05%, and 19.78 ± 6.43% after treatment with 0.3, 1, 3, and 10 μg/mL of MKE, respectively. These results show that MKE was non-toxic to RAW264.7 cells.

### 3.6. MKE Downregulates Osteoclast Differentiation-Related Gene Expression in RANKL-Stimulated RAW264.7 Cells

RANKL treatment increased the NFATc1, MMP9, TRAP, and Cathepsin K gene expression involved in osteoclast differentiation in RAW264.7 cells. Treatment with MKE at concentrations of 1, 3, and 10 μg/mL reduced the expression of these genes (Figure 4D). Specifically, MKE reduced *NFATc1* expression by 25.70 ± 3.08%, 30.13 ± 4.45%, and 31.86 ± 1.71%, respectively, compared to the group treated with RANKL alone. Similarly, *MMP9* expression decreased by 52.82 ± 10.44%, 66.80 ± 2.26%, and 71.48 ± 3.62%, respectively, while *TRAP* expression was reduced by 24.51 ± 4.22%, 25.74 ± 1.40%, and 22.75 ± 0.26% after treatment with 1, 3, and 10 μg/mL MKE, respectively, compared to treatment with RANKL alone. Additionally, Cathepsin K expression decreased by 38.70 ± 3.74% after treatment with 10 μg/mL of MKE.

### 3.7. MKE Downregulates Osteoclast Differentiation-Related Protein Expression in RANKL-Stimulated RAW264.7 Cells

The inhibitory effect of MKE on the RANKL-induced expression of genes involved in osteoclast function was confirmed via Western blotting (Figure 4E). When RAW264.7 cells were treated with RANKL, the protein expression of NFATc1, Cathepsin K, and TRAP was notably (^##^ *p* < 0.01) increased. Treatment with 3 and 10 μg/mL MKE reduced NFATc1 expression by 28.30 ± 3.10% and 34.93 ± 3.86%, respectively. In addition, Cathepsin K expression was reduced by 27.08 ± 4.18%, 65.58 ± 2.58%, and 83.38 ± 2.44%, while TRAP protein expression decreased by 58.63 ± 5.34%, 68.59 ± 4.71%, and 79.15 ± 8.71% after treatment with 1, 3, and 10 μg/mL MKE, respectively, compared to the group treated with RANKL only.

### 3.8. MKE Downregulates TRAF6 and MAPK Protein Expression in RANKL-Stimulated RAW264.7 Cells

**Figure 5:**
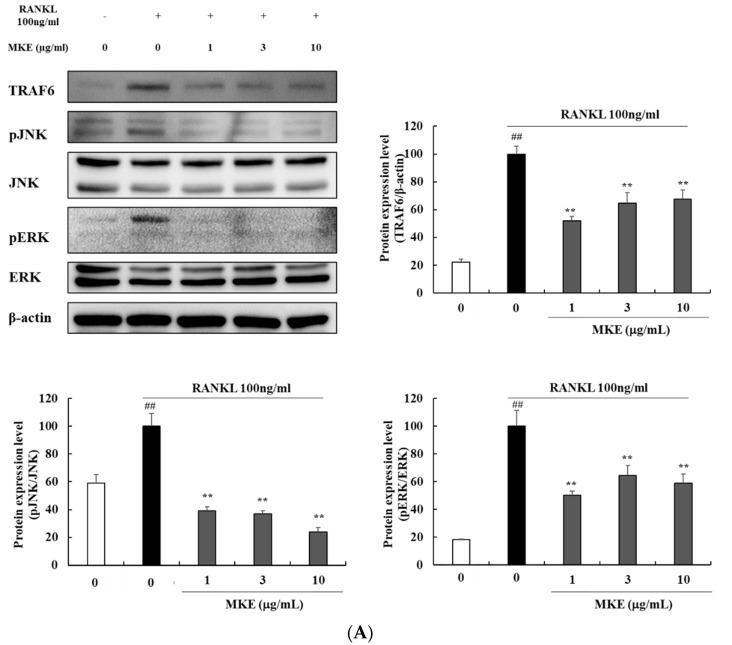

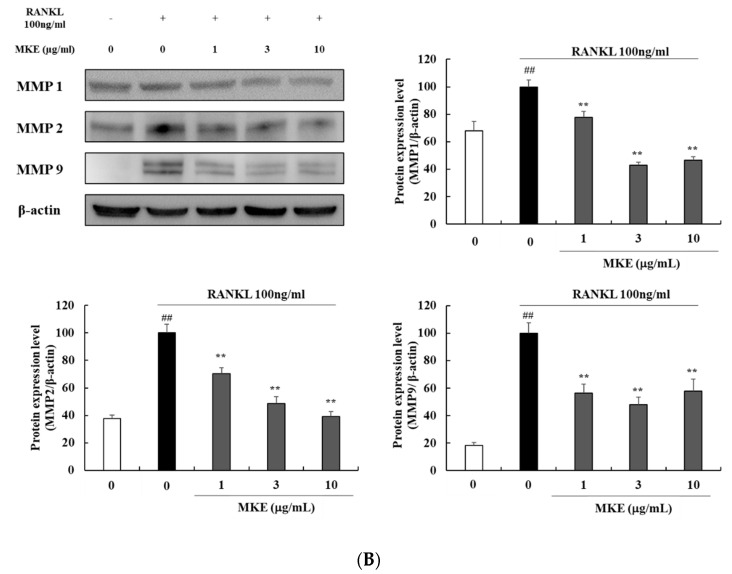
Effects of *Magnolia kobus* extract (MKE) (1, 3, or 10 μg/mL) treatment on the production of (**A**) TRAF6 and MAPK factors and (**B**) MMP proteins in RANKL-stimulated RAW264.7 cells. MKE and RANKL (100 ng/mL) were added to the cells, except for the vehicle control group, and incubated at 37 °C for 30 min or 5 days. The protein expression levels of TRAF6, pJNK, JNK, pERK, ERK, MMP-1, MMP-2, and MMP-9 were detected using a Western blot assay. The protein band density was measured using ImageJ software. Each experiment was repeated three times (*n* = 3) and each value was normalized to that of β-actin. Values represent the means ± standard deviation. ^##^ *p* < 0.01 vs. the vehicle control group; ** *p* < 0.01 vs. the RANKL-activated control group.

The effects of MKE on osteoclast differentiation were investigated by analyzing the protein expression of TRAF6 and MAP kinases to understand the underlying molecular mechanisms involved (Figure 5A). Western blot analysis was used to assess the phosphorylation of JNK and ERK, which are MAPK-related molecules. RANKL stimulation increased TRAF6 protein expression and phosphorylated JNK and ERK. However, MKE treatment reduced TRAF6 protein expression by 47.88 ± 3.19%, 35.21 ± 7.47%, and 32.26 ± 6.46% at concentrations of 1, 3, and 10 μg/mL, respectively. Furthermore, MKE treatment at 1, 3, and 10 μg/mL resulted in a decrease in the pJNK/JNK ratio by 60.71 ± 2.84%, 63.17 ± 2.34%, and 75.95 ± 3.21%, respectively. The ratio of pERK/ERK was also reduced by 49.73 ± 2.76%, 35.49 ± 7.05%, and 40.91 ± 6.58% after treatment with MKE at concentrations of 1, 3, and 10 μg/mL, respectively.

### 3.9. MKE Downregulates MMP Protein Expression in RANKL-Stimulated RAW264.7 Cells

To investigate the effect of MKE on the inhibition of matrix degradation, the protein expressions of MMP-1, -2, and -9 were analyzed using Western blotting (Figure 5B). The protein expressions of MMP-1, -2, and -9 were significantly increased after RANKL treatment. However, the RANKL-induced increase in MMP-1 expression decreased by 22.33 ± 4.29%, 57.00 ± 2.20%, and 53.63 ± 2.82% in the MKE 1, 3, and 10 µg/mL treatment groups, respectively, while MMP-2 expression was reduced by 29.52 ± 4.34%, 51.41 ± 5.15%, and 60.74 ± 3.51%, respectively. Furthermore, MMP-9 expression decreased by 43.48 ± 6.36%, 51.85 ± 5.42%, and 42.25 ± 8.61% in the groups treated with 1, 3, and 10 µg/mL MKE, respectively, compared to the RANKL-treated group.

## 4. Discussion

Periodontal disease, which is characterized by alveolar bone loss, is caused by periodontopathogenic bacteria, and its prevention is the most important clinical challenge. Periodontitis can lead to tooth loss through stages of gingival and bone tissue destruction, beginning with an inflammatory response [8,34]. In a previous study, we confirmed the inhibitory effect of MKE on the expression of inflammatory cytokines and MMPs through the TLR4-TAK1 signaling pathway in LPS-induced mouse macrophages [33]. In this study, we investigated the preventive effect of MKE on periodontitis by examining the inflammation and ECM degradation mechanisms of gingival fibroblasts and the expression of bone resorption-related factors in osteoclasts.

Periodontitis begins with an inflammatory response caused by bacteria that destroys gingival tissue. Pg is a representative connective organism, and LPS is its main virulence factor. Pg enters the connective tissue and stimulates cells, whereas LPS induces an inflammatory response. The most abundant human gingival fibroblasts in the periodontal connective tissue play an important role in the maintenance and damage of periodontal tissue and in the inflammatory response [35,36]. TLR4 combined with LPS has been shown to increase cytokine expression via the NF-κB pathway. IL-1 and IL-6 play key roles in the early phase of periodontitis and are detected at high levels in periodontal lesions. IL-1β increases the collagenolytic activity of fibroblasts, while IL-6 participates in osteoclast differentiation and bone resorption. TNF-α also promotes the HMGB1-induced production of osteoclastogenic cytokines involved in osteoclastogenesis [12,35,36,37]. In addition, these cytokines increase adhesion molecules such as VCAM-1 and ICAM-1 expression. Increased VCAM-1 and ICAM-1 expression recruits inflammatory cells to promote an inflammatory response and is involved in the progression of chronic inflammation and periodontitis [14,17].

LPS-PG-induced gingival fibroblasts activate the TLR4/NF-κB pathway and significantly increase the expression of cytokines and genes such as *HMGB1*, *ICAM-1*, and *VCAM-1*. However, MKE treatment significantly reduced the protein expression of IL-1β, IL-6, and TNF-α and related genes such as *HMGB1*, *ICAM-1*, and *VCAM-1* by inhibiting the TLR4/NF-κB pathway. Therefore, MKE treatment alleviates periodontitis progression by suppressing the initial inflammatory response and regulating the expression of osteoclast-related factors.

Fibroblasts secrete MMPs and are involved in the destruction of the ECM and bone collagen and play an important role in connective tissue destruction. TIMPs are proteins that inhibit MMP activity; when the balance between MMPs and TIMPs is disrupted, the ECM is destroyed, and periodontitis occurs. Therefore, regulating the expression of MMPs and TIMPs in LPS-induced human gingival fibroblasts can prevent gingival tissue destruction and inhibit periodontitis progression [35,36]. In our study, MKE treatment significantly suppressed the protein expression levels of MMP-1, -3, -8, -9, and -13 and increased the protein expression levels of TIMP-1 and -2 induced by LPS-PG treatment.

The hallmark of periodontitis is alveolar bone loss; osteoclasts, which are bone resorptive cells, are involved in bone destruction. LPS plays an important role in bone resorption and activates osteoclast formation and differentiation by stimulating the secretion IL-1, IL-6, and TNF-α cytokines. In addition, cell fusion is a critical stage during osteoclast formation for acquiring bone resorption activity. Fusion to multinucleated osteoclasts occurs after RANKL treatment. In this study, we investigated whether multinucleated osteoclast formation was significantly reduced upon treatment with MKE. Previous studies have reported that osteoclast activation was inhibited by reducing the secretion of IL-1β, IL-6, and TNF-α in LPS-induced HGF-1 cells [34,37,38,39].

RANKL, which plays a pivotal role in the survival, differentiation, multi-nucleation, and activation of osteoclasts, activates TRAF6 by binding to RANK. TRAF6 activation promotes osteoclast differentiation by stimulating the MAPK signaling pathway. JNK plays a vital role in osteoclast differentiation, whereas ERK plays a functional role in osteoclast differentiation and survival. In a previous study, MKE inhibited osteoclast differentiation by blocking the increases in TRAF6, JNK, and ERK expression induced by RANKL treatment [40].

The phosphorylation of MAPKs by RANKL activates NFATc1, increasing the expression of the osteoclast-specific genes *CTSK* and *TRAP*. Treatment with MKE significantly reduced the protein expression of NFATc1 and CTSK, which increased with RANKL treatment. Additionally, TRAP activity and gene and protein expression levels were significantly reduced with MKE treatment. NFATc1 also upregulates MMP expression. MMP-9 is an enzyme that is expressed during bone resorption in osteoclasts and is significantly downregulated upon MKE treatment. The increased protein expression of MMP-1 and MMP-2 upon RANKL treatment was also significantly suppressed. These results demonstrate that MKE treatment blocks bone resorption and periodontal destruction by suppressing osteoclast differentiation [41,42,43].

**Figure 6:**
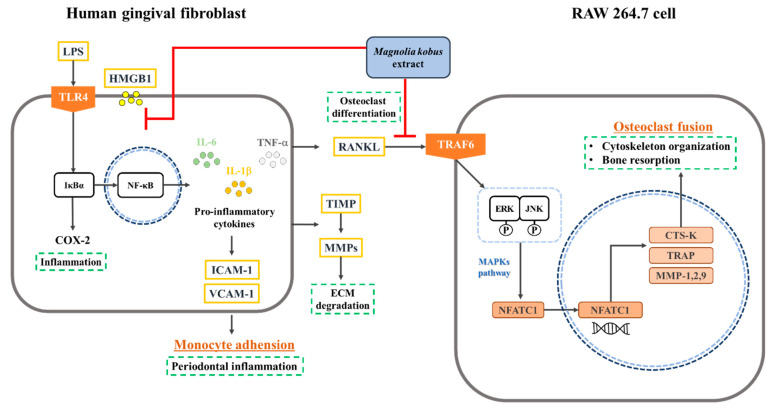
Anti-periodontitis mechanism of *Magnolia kobus* extract (MKE) in human gingival fibroblasts and RAW264.7 cells.

In this study, MKE suppressed bone resorption by reducing early cytokine expression and inhibiting osteoclast differentiation by destroying gingival tissue (Figure 6). These effects were modulated by downregulating the TLR4/NF-κB pathway in LPS-induced HGF-1 cells, inhibiting IL-1β, IL-6, and TNF-α cytokines expression. Decreased cytokine expression also inhibits the secretion of osteoclastogenic cytokines and the progression of the inflammatory response by inhibiting the expression of HMGB1, ICAM-1, and VCAM-1. Additionally, MKE treatment regulated MMP and TIMP expression to reduce ECM degradation.

Moreover, MKE treatment downregulated the TRAF6/MAPK pathway in RANKL-stimulated osteoclasts, decreased NFATc1 expression, and inhibited osteoclast-specific gene expression. These findings suggest that MKE can be developed as a preventive and therapeutic agent by regulating typical clinical responses to periodontal diseases. To investigate the potential of MKE as a treatment for periodontal disease, we have planned additional in-vivo experiments. The upcoming in vivo study aims to assess MKE’s effectiveness in reducing inflammation, inhibiting ECM degradation, suppressing MMPs expression, and verify if MKE exhibits a signal transduction pathway similar to that observed in the in-vitro study.

## Figures and Tables

**Table 1:** Primer sequences used in this study.

Gene	Sequence (5′-3′)	Conditions
*HMGB1*	F: CGC CAT GAG AAC TTC CTA CC R: CAC TTG GCC TTC CCT CTG TA	40 cycles at the following conditions: 94 °C for 40 s, 55 °C for 40 s, and 72 °C for 1 min
*ICAM-1*	F: CGT GCC GCA CTG AAC TGG AC R: CCT CAC ACT TCA CG TCA CCT	
*VCAM-1*	F: ATT GGG AAA AAC AGA AAA GAG R: GGC AAC ATT GAC ATA AAG T	
*TIMP-1*	F: TTC GTG GGG ACA CCA GAA GTC AAC R: TGG ACA CTG TGC AGG CTT CAG TTC	
*TIMP-2*	F: AAG CGG TCA GTG AGA AGG AGT GG R: CCT TGG AGG CTT TTT TGC AGT TG	
*GAPDH*	F: TGA AGG TCG GAG TCA ACG GAT TTG GT R: CAT GTG GGC CAT GAG GTC CAC CAC	
*NFATc1*	F: CCGTTGCTTCCAGAAAATAACA R: TGTGGGATGTGAACTCGGAA	40 cycles at the following conditions: 95 °C for 5 s, 60 °C for 20 s, and 72 °C for 20 s
*TRAP*	F: CTGGAGTGCACGATGCCAGCGACA R: TCCGTGCTCGGCGATGGACCAGA	
*GAPDH*	F: ACCCAGAAGACTGTGGATGG R: CACATTGGGGGTAGGAACAC	
*MMP9*	F: GCCCTGGAACTCACACGACA R: TCCGTGCTCGGCGATGGACCAGA	35 cycles at 95 °C for 5 s and 60 °C for 20 s
*Catehpsin K*	F: CAGCAGAACGGAGGCATTGA R: CCTTTGCCGTGGCGTTATAC	

## Data Availability

Not applicable.
